# Dietary and Fluid-Intake Habits in Patients with Various Types of Urinary Incontinence: A Preliminary Study

**DOI:** 10.3390/nu18193127

**Published:** 2026-09-23

**Authors:** Aleksandra Piątek, Gabriela Kołodyńska, Krzysztof Nowak, Jarosław Drapała, Felicja Fink-Lwow, Waldemar Andrzejewski, Maciej Zalewski

**Affiliations:** 1 Department of Gynecology, Independent Public Health Care Center of the Ministry of the Interior and Administration in Wroclaw, 50-233 Wroclaw, Poland; 2 Faculty of Physiotherapy, Wroclaw University of Health and Sport Sciences, 51-612 Wroclaw, Poland; 3 Faculty of Information and Communication Technology, Wroclaw University of Science and Technology, 50-370 Wroclaw, Poland

**Keywords:** nutrition, dietary habits, urinary incontinence, overactive bladder, fluid intake, PFDI-20

## Abstract

**Background/Objectives:** Urinary incontinence (UI) is common in women, particularly during and after the menopausal transition, and may both influence and be influenced by dietary and fluid-intake behaviours. This exploratory study described self-reported dietary and fluid-intake habits in women with different UI subtypes and explored their associations with pelvic floor symptom burden and self-reported fluid restriction. Energy and nutrient intake was not quantified. **Methods:** This exploratory, cross-sectional, single-centre study included 87 women aged 18–84 years with stress (SUI; *n* = 23), urgency (UUI; *n* = 21), or mixed (MUI; *n* = 43) UI, who reported UI at least weekly. Participants completed a custom dietary and fluid-intake questionnaire, the Incontinence Quality of Life Questionnaire (I-QOL), and the Pelvic Floor Distress Inventory-20 (PFDI-20). Associations were assessed using rank correlations and proportional-odds ordinal logistic regression, with Benjamini–Hochberg correction used to address multiplicity in exploratory item-level analyses. **Results:** No individual diet-PFDI association remained statistically significant after Benjamini–Hochberg correction across the complete 100-test matrix. In the adjusted model, an urgency component was associated with greater self-reported fluid restriction (adjusted cumulative OR = 2.92; 95% CI, 1.18–7.20), whereas each 10-year increase in age was associated with lower odds of greater restriction (adjusted cumulative OR = 0.68; 95% CI, 0.51–0.92). **Conclusions:** Greater self-reported fluid restriction was independently associated with an urgency component and with younger age, whereas no individual dietary or fluid-intake item remained statistically significant after correction for multiple comparisons. Given the potential risk of the practice, these findings support asking women with urgency symptoms about fluid restriction during clinical consultations. Prospective studies using a validated quantitative dietary instrument are needed to determine whether dietary and fluid-intake habits affect pelvic floor symptom burden.

## 1. Introduction

Urinary incontinence affects more than 50% of menopausal women worldwide. Incontinence comprises several types of varying aetiology [1]. The International Continence Society (ICS) distinguishes five types of urinary incontinence, with a predominant prevalence of stress urinary incontinence (SUI)—50–75%, mixed urinary incontinence (MUI)—up to 30% and urge urinary incontinence (UUI)—up to 20%. Other types are functional and overflow incontinence [1].

While SUI is associated with urethral hypermobility due to deterioration of the quality of tissues supporting the urethra or intrinsic urethral sphincter deficiency, UUI is associated with detrusor overactivity. With age, many patients develop a mixed form containing both types—MUI [2]. The symptoms usually aggravate with age owing to tissue laxity and hormonal changes [3]. Spinal degenerative processes may also trigger sacral nerve irritation, resulting in overactive bladder (OAB) symptoms [4].

The risk factors across all types predominantly included carbohydrate metabolism disorders, a history of vaginal delivery, a sedentary lifestyle and being overweight or obese [1]. Sleep disorders, depression and abnormal autonomic nervous system activity may also trigger or worsen the symptoms [5].

As urinary incontinence affects many areas of daily activity, it may also influence dietary routine and fluid intake, and dietary behaviour may in turn modify symptom burden. Weight loss and dietary modification are well established as means of reducing the severity of UI symptoms [6], and adequate nutrition is associated with lower systemic inflammation and autonomic nervous system homeostasis [5]. Conversely, many women with urgency symptoms deliberately restrict fluid intake in an attempt to reduce leakage, which is a coping behaviour. It might lead to urine concentration, aggravated urgency, and a higher risk of urinary tract infections and constipation [4,7]. The aim of this exploratory study was to describe self-reported dietary and fluid-intake behaviour in women with different urinary-incontinence types. Also, we aimed to examine the relation of the behaviour and pelvic floor symptom severity. The primary question was specified before analysis: whether women with an urgency component would report greater fluid restriction than those without, independent of age, diabetes, body mass index and total fluid intake. All other analyses reported here are secondary and exploratory and are intended to generate hypotheses for future work rather than to test them.

## 2. Materials and Methods

### 2.1. Study Design

We conducted a single-centre, cross-sectional study of women presenting with UI to a urogynaecology outpatient clinic. The study is in accordance with the Strengthening the Reporting of Observational Studies in Epidemiology (STROBE) guidance [8]; the completed STROBE checklist is provided in Supplementary Material as Table S1.

The analysis was a secondary exploratory investigation of a clinical questionnaire dataset; the primary hypothesis and supporting covariate set were specified before the final analysis.

### 2.2. Setting and Participants

Consecutive women evaluated for pelvic floor complaints during the study period (from January 2025 to March 2026) were invited. The inclusion criteria for the present analysis were female sex, age ≥ 18 years, urinary incontinence and voluntary consent to participate. The exclusion criteria were male sex, age < 18 years, a current genitourinary infection and lack of consent. Participation was voluntary and anonymous. Informed consent was obtained from all participants. The study followed the Declaration of Helsinki [9]. Data were collected in the Department of Gynecology Independent Public Health Care Center of the Ministry of the Interior and Administration in Wroclaw between January 2025 and March 2026.

This study involved 87 women aged 18–84 years reporting urinary incontinence episodes more than once a week. The study was approved by the Bioethics Committee of the Medical University in Wroclaw (decision no. 346/2024, dated 23 May 2024). No personally identifiable data were used and participants were referenced only by a surrogate identifier.

Participant flow is summarised in Figure 1. During the recruitment period, 184 consecutive women attending the Department of Gynecology were invited to participate; 23 declined. A total of 161 women consented and completed the single study visit and formed the parent cohort of this study: 87 reported urinary incontinence at least once weekly; 53 had symptomatic pelvic organ prolapse without urinary incontinence; and 21 were continent women recruited as a comparison group. The present report is restricted to the 87 women with urinary incontinence; the remaining 74 women are reported separately. Because all exposures and outcomes were recorded at that single visit, there was no follow-up interval and consequently no loss to follow-up; no participant was withdrawn after data collection. Isolated missing responses were confined to exploratory bowel items (*n* = 86 for those analyses), whereas every variable entered into the regression models was complete for all 87 participants.

### 2.3. Variables and Clinical Assessment

UI type was assigned clinically from medical history. Patients were assigned to three groups based on their medical history. In cases of clinical uncertainty, urodynamic studies and bladder diaries were used.

Women reporting stress-related leakage alone were classified as SUI (*n* = 23), those reporting urgency-related leakage alone as UUI (*n* = 21) and those reporting both as MUI (*n* = 43). The primary exposure was the presence of an urgency component, defined as clinically documented urinary urgency with or without urgency incontinence. On this definition an urgency component was present in 64 women and absent in 23. These two classifications coincide exactly: the urgency-positive group comprises UUI and MUI, and the urgency-negative, SUI.

The primary outcome was self-reported fluid restriction (I-QOL item 1.8; scored 1–5, higher = greater restriction). Prespecified covariates included age, diabetes, BMI and fluid-intake score. Supporting variables included self-reported dietary/fluid habits and pelvic floor symptom severity (PFDI [10]); quality of life was assessed with the I-QOL [11]. Reported fluid intake was recorded as an ordinal category and not as a measured volume: participants selected their usual daily intake from response options. Because intake was categorical—defined in glasses of approximately 250 mL (from one glass or less to more than six glasses, coded 1 to 4)—this comparison is approximate, and no individual intake volume was derived.

Dietary and fluid habits were captured with a custom, non-validated frequency questionnaire; our framing is therefore restricted to habits, not quantified nutrient or energy intake. Years since last menstruation was calculated as the interval in completed years between the reported date of the final menstrual period and the study visit. Premenopausal women were coded as 0 years, and menopausal status was retained as a separate binary variable so that premenopausal women were not conflated with recently postmenopausal women.

Comorbidities were determined on the medical history and documentation. Diabetes mellitus was recorded when a participant had a physician diagnosis or was receiving glucose-lowering therapy, or a fasting plasma glucose ≥ 7.0 mmol/L (126 mg/dL) (WHO/ADA criteria) [12,13]. Hypertension was defined as a documented diagnosis or ongoing antihypertensive treatment, or repeated office blood pressure ≥ 140/90 mmHg (ESC/ESH criteria) [14]. Body mass index (BMI) was calculated as weight in kilograms divided by the square of height in metres (kg/m^2^) from measurements taken at the study visit.

The questionnaire was developed by the investigators and administered in Polish, the first language of all participants, as a part of a five-part clinical form (see Table S2 in Supplementary Material). It was self-completed on the day of the study visit, and checked for completeness in return. Table S2 shows isolated missing responses in some exploratory bowel item analyses (*n* = 86), although all regression-model variables were complete (*n* = 87). The dietary and fluid items are phrased as frequency questions reporting usual consumption. A custom-designed questionnaire was administered alongside two validated instruments. Each dietary item used seven ordered response categories running from several times a day to never, coded 1 to 7 as collected, so that higher scores denote less or more frequent intake. The instrument was not designed as a dietary assessment tool. All patients underwent a gynaecological examination and ultrasound assessment on the same day as they completed the questionnaires. The impact of incontinence on quality of life was assessed using the Incontinence Quality of Life Questionnaire (I-QOL). Additionally, the Pelvic Floor Distress Inventory (PFDI-20) was used, which comprises three subscales: the Pelvic Organ Prolapse Distress Inventory (POPDI-6) for prolapse-related complaints; the Colorectal-Anal Distress Inventory (CRADI-8) for defecation disorders; and the Urinary Distress Inventory (UDI-6) for urinary symptoms. In the custom-designed questionnaire, patients were asked to grade the severity of various symptoms—urgency, stress incontinence, nocturia, stool loss, flatus loss and abdominal pain—from 0 (not severe at all) to 10 (most bothersome), each severity item being accompanied by a companion item recording how often the symptom occurred, using the same seven categories as the dietary items.

### 2.4. Statistical Analysis

A total of 144 variables were collected, of which 45 were included in the analyses. The analyses were organised hierarchically. The primary inferential analysis evaluated whether the presence of an urgency component was associated with greater self-reported fluid restriction (I-QOL item 1.8) after adjustment for age, diabetes, BMI, and reported fluid intake. The urgency coefficient was the primary inferential target, whereas the remaining variables in this model were adjustment covariates. Analyses involving individual dietary/fluid variables and PFDI items, SUI-VAS severity, grouped OAB-VAS severity, and smoking status were considered secondary and exploratory. Given the sample size of 87 patients and the number of exploratory associations examined, these secondary findings were interpreted as hypothesis-generating.

The item-level diet/fluid-PFDI analysis comprised 100 possible comparisons between five dietary/fluid variables and the 20 individual PFDI items. To account for multiplicity across the full exploratory analysis, Benjamini–Hochberg correction was applied to the complete 100-test matrix. The eight associations displayed in Figure 2 were selected for descriptive presentation and are treated as exploratory illustrations rather than as a separate confirmatory testing family. For transparency, Benjamini–Hochberg-adjusted *p*-values calculated within this eight-association subset are also reported descriptively, but they were not used as the basis for confirmatory inference. The analyses using alternative OAB-VAS cut-points (≥8, ≥9, and 10) were conducted post hoc as exploratory sensitivity analyses; their *p*-values were not adjusted for multiplicity and were not used to define the primary conclusions. No multiplicity correction was applied across the separate regression models, because these represented distinct analyses rather than a single testing family. Within the SUI-VAS model, Benjamini–Hochberg correction was applied across the seven predictors, whereas the post hoc OAB-VAS threshold analyses were left unadjusted and interpreted as exploratory.

Analyses were based on complete cases for the variables required by each analysis. No missing values were present in the variables used in the reported regression models, and all multivariable models therefore included 87 participants. Some exploratory questionnaire-item analyses contained isolated missing responses and were performed using available cases. No imputation was performed. Predictor sets were specified directly; no stepwise or significance-based variable selection was performed. Age and years since last menstruation were scaled per 10-year increment when reporting odds ratios. OAB-VAS was grouped into low (0–3), moderate (4–7), and high (8–10) categories for the exploratory ordinal model, while alternative OAB-VAS thresholds were examined only in post hoc exploratory sensitivity analyses.

Smoking status was not included in the multivariable model because only five participants were smokers, resulting in severe imbalance and a high risk of unstable coefficient estimates. Its association with OAB-VAS was therefore examined separately in an exploratory unadjusted analysis. Nevertheless, residual and unmeasured confounding could not be excluded. Thus, the observed associations may reflect true relationships, chance findings, or the influence of uncontrolled confounding factors.

No formal a priori sample-size calculation was performed. The sample size was determined by the number of consecutive eligible patients recruited during the study period rather than by a statistically defined target. To characterise the statistical sensitivity of the primary analysis, we additionally performed a simulation-based sensitivity analysis for the urgency coefficient in the proportional-odds model. With the available sample of 87 participants and a two-sided α of 0.05, the study had approximately 80% power to detect an adjusted cumulative odds ratio of approximately 3.5 for urgency status. This analysis was used to characterise the detectable effect size of the available sample and not as a retrospective justification of the sample size.

Bivariate associations were assessed primarily using Spearman’s rank correlation. Kendall’s tau-b was calculated as a complementary measure of ordinal association, particularly in the presence of tied observations. Bootstrap 95% confidence intervals were calculated for the correlation coefficients. Differences in continuous or ordinal variables across more than two ordered response categories were assessed using the Kruskal–Wallis test, whereas prespecified two-group comparisons were evaluated with the Mann–Whitney U test, with the rank-biserial correlation reported as an effect-size measure together with bootstrap confidence intervals. All tests were two-sided.

Correlation heat maps were constructed to visualise pairwise associations among candidate predictors and to screen for potential collinearity (Figure S1). Spearman’s rank correlation was used for non-binary pairs and binary-ordinal pairs, point-biserial correlation for binary-continuous pairs, and the phi coefficient for binary-binary pairs.

Multivariable associations of candidate predictors with ordered questionnaire-item responses were estimated using proportional-odds ordinal logistic regression and reported as adjusted cumulative odds ratios with 95% confidence intervals. Odds ratios above 1 indicated higher odds of being in a higher outcome category, according to the coding of the response variable. The parallel-lines assumption was assessed globally and for individual predictors using a Brant-type Wald diagnostic implemented in Python v3.14 (Python Software Foundation, Beaverton, OR, USA); numerically unstable assessments were classified as indeterminate.

All fitted ordinal and binary regression models were assessed for convergence. For the Brant-type diagnostic, assessments affected by numerical instability or rank deficiency of the contrast covariance matrix were classified as indeterminate rather than interpreted as evidence for or against the proportional-odds assumption.

## 3. Results

### 3.1. Participant Characteristics

The characteristics of the analysed group are summarised in Table 1. Among the patients, 56 of 87 (64.4%) were menopausal.

### 3.2. Dietary and Fluid-Intake Associations

To facilitate interpretation of the exploratory analyses presented in this subsection, nominal *p*-values represent unadjusted individual tests, whereas the BH-adjusted *p*-values reported for the eight selected diet-PFDI associations account only for multiplicity within that displayed subset. Accordingly, values below 0.05 in the nominal or subset-adjusted analyses should be interpreted as exploratory signals rather than statistically significant findings.

No association was found between BMI (body mass index) and the frequency of fruit or vegetable consumption. Higher BMI was weakly associated with more frequent sweets consumption, although the association was not statistically significant (Spearman’s ρ = −0.203; 95% CI [−0.393, 0.011]; unadjusted *p* = 0.059; BH-adjusted *p* = 0.073), and an inverse association was observed regarding the consumption of sugar in beverages (Spearman’s ρ = 0.270; 95% CI [0.051, 0.470]; unadjusted *p* = 0.011; BH-adjusted *p* = 0.046) (Figure 2a,b). No significant correlation was found between BMI and the frequency or severity of nocturia. Furthermore, nocturia parameters showed no association with total fluid intake.

Notably, there were no significant differences in fluid intake among the UUI, SUI and MUI groups. Similarly, no significant variation in fluid intake was found with respect to patients’ BMI or age.

Higher BMI was weakly associated with higher defecation frequency, although the association did not remain statistically significant after correction for multiple testing (Spearman’s ρ = −0.217; 95% CI [−0.426, 0.002]; unadjusted *p* = 0.044; BH-adjusted *p* = 0.073) (Figure 2c). Lower BMI was weakly associated with more constipation-type stool characteristics, although the association was not statistically significant (Spearman’s ρ = 0.193; 95% CI [−0.018, 0.395]; unadjusted *p* = 0.073; BH-adjusted *p* = 0.073), and no significant association was found between BMI and the Bristol Stool Scale overall (Figure 2d).

Neither sweets consumption of different types nor sugar use was associated with the frequency or consistency of stool; fluid intake was likewise unrelated to stool type or frequency.

Among the eight diet-PFDI associations highlighted in the initial analysis, seven had Benjamini–Hochberg-adjusted *p*-values below 0.05 when correction was restricted to those eight comparisons; the exception was the association between fruit consumption and involuntary loss of well-formed stool (PFDI A3; BH-adjusted *p* = 0.051). However, because these eight comparisons were not treated as a separate confirmatory testing family, multiplicity was additionally assessed across the complete matrix of 100 comparisons between the five dietary/fluid variables and the 20 individual PFDI items. Under this full-matrix correction, no individual diet-PFDI association remained statistically significant. The selected associations presented below and illustrated in Figure 2 should therefore be regarded as hypothesis-generating observations.

More frequent sugar use was associated with greater lower-abdominal pressure symptoms, as assessed by PFDI P1 (Spearman’s ρ = −0.252; 95% CI [−0.449, −0.045]; unadjusted *p* = 0.019; BH-adjusted *p* = 0.031) (Figure 2e–g). More frequent fruit consumption was associated with more frequent reports of a bulge or a sensation of something falling in the vaginal area (PFDI P3) (Spearman’s ρ = −0.323; 95% CI [−0.489, −0.123]; unadjusted *p* = 0.002; BH-adjusted *p* = 0.018) (Figure 2e–g). The feeling of incomplete bladder emptying (PFDI P5) was found to correlate with sweets consumption (Spearman’s ρ = −0.269; 95% CI [−0.453, −0.045]; unadjusted *p* = 0.012; BH-adjusted *p* = 0.031) (Figure 2e–g).

More frequent fruit consumption was weakly associated with higher PFDI A3 scores, reflecting greater bother related to involuntary loss of well-formed stool, although the association did not reach the threshold for statistical significance (Spearman’s ρ = −0.210; 95% CI [−0.394, −0.009]; unadjusted *p* = 0.051; BH-adjusted *p* = 0.051) (Figure 2h).

Conversely, more frequent sweetening of beverages was associated with lower scores for both involuntary loss of well-formed stool (PFDI A3; Spearman’s ρ = 0.248; 95% CI [0.056, 0.425]; unadjusted *p* = 0.021; BH-adjusted *p* = 0.031) and involuntary loss of loose stool (PFDI A4; Spearman’s ρ = 0.263; 95% CI [0.075, 0.435]; unadjusted *p* = 0.014; BH-adjusted *p* = 0.031) (Figure 2i,j).

Lower fluid consumption was weakly associated with flatal incontinence (PFDI A5) (Spearman’s ρ = 0.243; 95% CI [0.036, 0.440]; unadjusted *p* = 0.023; BH-adjusted *p* = 0.031) (Figure 2k).

Patients with urinary leakage related to coughing, sneezing or laughing (PFDI U3) reported less frequent fruit consumption (Spearman’s ρ = 0.225; 95% CI [0.017, 0.412]; unadjusted *p* = 0.036; BH-adjusted *p* = 0.041) (Figure 2l).

### 3.3. Factors Associated with Fluid Restriction

To examine factors associated with reported fluid restriction, we considered urgency status, age, diabetes, body mass index and reported fluid consumption as explanatory variables, with the I-QOL 1.8 fluid-restriction item as the dependent variable. Because this outcome was ordinal and ranged from 1 to 5, a proportional-odds ordinal logistic regression model with a logit link was fitted to estimate mutually adjusted associations. Results were expressed as adjusted cumulative odds ratios with 95% confidence intervals, with values above 1 indicating higher odds of being in a higher fluid-restriction category. Univariable and multivariable estimates are presented in Table 2.

In the adjusted ordinal logistic regression model, urgency status and age were the only variables significantly associated with reported fluid restriction. The urgency was associated with higher odds of reporting greater fluid restriction (adjusted cumulative OR = 2.92; 95% CI [1.18, 7.20]; *p* = 0.020), whereas each 10-year increase in age was associated with lower odds (adjusted cumulative OR = 0.68; 95% CI [0.51, 0.92]; *p* = 0.011). The proportional-odds assumption was not rejected (global Brant-type Wald test, *p* = 0.760). Diabetes, body mass index and fluid consumption were not independently associated with reported fluid restriction.

Because reported fluid consumption had only three observed levels, we additionally performed a sensitivity analysis in which this variable was entered into the ordinal logistic regression model as a categorical rather than a linear predictor. The association between the urgency component and reported fluid restriction remained consistent and statistically significant in this model (adjusted cumulative OR = 2.72; 95% CI [1.09, 6.81]; *p* = 0.032), supporting the robustness of the primary analysis with respect to the coding of fluid consumption. In addition, inspection of the outcome distribution showed that all five response categories were represented in both urgency-positive and urgency-negative groups; the highest fluid-restriction category was reported by 39.1% of patients with an urgency component and 17.4% of those without. Pairwise correlations among the candidate predictors were low (maximum absolute correlation r = 0.204), providing no indication of substantial pairwise collinearity.

Although participants reported changing the timing of fluid intake or avoiding fluids before important events, they did not report substantial changes to their overall daily routines.

### 3.4. Factors Associated with SUI Severity

To examine factors associated with subjectively perceived stress urinary incontinence severity, we considered years since last menstruation, fruit consumption, vegetable consumption, sweets consumption, sweetening of beverages, body mass index and number of deliveries as explanatory variables, with SUI-VAS (a 0–10 visual analogue scale of self-perceived stress-incontinence severity) as the dependent variable. Because SUI-VAS was an ordered 0–10 scale and showed a ceiling effect with sparse counts in some categories, a proportional-odds ordinal logistic regression model with a logit link was used to estimate adjusted associations with the odds of reporting higher symptom severity. Although the computed global Brant-type statistic corresponded to a nominal *p* = 0.031, the contrast covariance matrix was rank-deficient, preventing reliable interpretation of the global diagnostic. The proportional-odds diagnostic was therefore considered indeterminate, and the adjusted cumulative odds ratios should be interpreted cautiously as exploratory common-effect summaries across cumulative outcome thresholds.

Adjusted cumulative odds ratios above 1 indicated higher odds of being in a higher SUI-VAS category, whereas values below 1 indicated lower odds of being in a higher category. All candidate predictors were entered simultaneously into the multivariable ordinal logistic regression model. Table 2 provides the corresponding univariable and multivariable estimates for all predictors included in this model.

In the proportional-odds model, each 10-year increase in years since last menstruation was inversely associated with subjectively perceived stress urinary incontinence severity (adjusted cumulative OR = 0.62; 95% CI [0.42, 0.91]; nominal *p* = 0.015), although this association did not remain statistically significant after Benjamini–Hochberg correction across the seven predictors in the model (BH-adjusted *p* = 0.102). Fruit consumption score showed a borderline, model-dependent positive association with SUI-VAS severity, although the association did not meet the conventional threshold for statistical significance (adjusted cumulative OR = 1.35; 95% CI [1.00, 1.82]; nominal *p* = 0.051; BH-adjusted *p* = 0.180); because higher scores indicated less frequent fruit consumption, this result was not interpreted as evidence of a directional dietary effect. Vegetable consumption, sweets consumption, sweetening of beverages, body mass index and number of deliveries were not statistically significant predictors of the SUI-VAS. No predictor remained statistically significant after Benjamini–Hochberg correction across the seven predictors. Given the indeterminate proportional-odds diagnostic described above, these estimates should be interpreted cautiously as common-effect summaries across cumulative outcome thresholds.

### 3.5. Factors Associated with OAB Severity and UI Subtype

To examine factors associated with subjectively perceived OAB symptom severity, we considered vegetable consumption, years since last menstruation, pelvic organ prolapse, body mass index, fruit consumption, abdominal surgery and number of deliveries as explanatory variables, with grouped OAB-VAS severity as the dependent variable. Because OAB-VAS was an ordered 0–10 scale with sparse counts in some categories and visible floor and ceiling effects, it was grouped into low (0–3), moderate (4–7) and high (8–10) severity categories and analysed using a proportional-odds ordinal logistic regression model with a logit link. In the fully adjusted model, none of the analysed variables was significantly associated with grouped OAB-VAS severity; the strongest, although non-significant, positive trend was observed for vegetable consumption (adjusted cumulative OR = 1.37, 95% CI [0.87, 2.16]; *p* = 0.177). The proportional-odds assumption was not rejected (global Brant-type Wald test, *p* = 0.304).

Neither fruit nor vegetable consumption was significantly associated with grouped OAB-VAS severity in the multivariable model. Smoking status was not included in the multivariable model because only five participants were smokers. In a separate exploratory unadjusted analysis, smokers had higher OAB-VAS scores than non-smokers (median 10 [IQR 9–10] vs. 7 [IQR 3–9]; Mann–Whitney U test, *p* = 0.034). However, because only five participants (5.7% of the sample) were smokers, the observed association was based on a very small and highly imbalanced group and may be unstable and susceptible to confounding.

In post hoc sensitivity analyses using alternative binary definitions of severe OAB-VAS, higher vegetable-frequency consumption scores showed nominal associations with greater adjusted odds of OAB-VAS ≥ 9 (adjusted OR = 1.90; 95% CI [1.29, 2.80]; *p* = 0.001) and an OAB-VAS score of 10 (adjusted OR = 1.72; 95% CI [1.16, 2.54]; *p* = 0.007), but not with OAB-VAS ≥ 8. Fruit consumption was associated with severity only at the ≥9 cut-point (adjusted OR = 1.50; 95% CI [1.04, 2.14]; *p* = 0.028). No other predictors showed nominal associations across the tested cut-points. Because these thresholds were examined post hoc and the *p*-values were not adjusted for multiplicity, these findings should be interpreted only as exploratory and sensitive to outcome definition.

Urinary distress (UDI-6) was highest in the MUI group (mean 65.1 vs. 43.8 in SUI and 47.4 in UUI; *p* < 0.001), and prolapse distress (POPDI-6) was numerically greater in the UUI and MUI groups than in SUI, with a significant overall difference across subtypes (*p* = 0.020) (Table 1). No statistically significant differences in the measured dietary variables were observed across subtypes (all *p* > 0.054). Thus, subtype-related differences were apparent in symptom burden but not in the assessed dietary characteristics. Full regression estimates for this model are shown in Table 2.

## 4. Discussion

In this exploratory cross-sectional study of 87 women with urinary incontinence, the presence of an urgency component was associated with greater self-reported fluid restriction after adjustment, with approximately threefold adjusted odds of reporting a higher restriction category, while older age was associated with less restriction. The item-level associations between dietary/fluid variables and pelvic floor symptoms were weak. Of the eight diet-PFDI associations highlighted for descriptive presentation, seven had Benjamini–Hochberg-adjusted *p*-values below 0.05 when correction was restricted to those eight comparisons. However, after Benjamini–Hochberg correction across all 100 item-level comparisons between the five dietary/fluid variables and the 20 individual PFDI items, no individual diet-PFDI association remained statistically significant. These item-level associations are discussed as questions for future work rather than as definitive findings.

Our observations align with data showing fluid restriction and greater caffeine avoidance among people with urgency [7] and extend to previous work identifying fluid-intake scheduling [1]. Deliberate adjustment of fluid intake has been reported as a risk-stratification axis in postmenopausal women [1], which in our study was found to be less relevant when compared to younger patients. This modifiable behaviour may entail consequences associated with symptom aggravation due to altered bowel functioning and a higher risk of urinary tract infection [4]. Our cross-sectional study cannot show that restriction worsens symptoms or the causality direction of factors, but enables us to identify the group of patients in whom behaviour is common enough to be worth eliciting at consultation.

Concerning the amount of fluid consumed, patients are advised to drink at least 30 mL of fluid per kilogram of body weight, or at least 2 L per day. In the study of Pavlovska, 88% of patients with SUI did not drink enough fluid compared with 12% of continent women. In that study, a similar pattern of conscious fluid reduction was noted in 59% of women, who reduced intake before journeys [1]. In our sample, most women reported intake in the highest categories, which may reflect differences in how intake was ascertained as much as genuine differences in behaviour.

The inverse association with age was not anticipated. This finding is only partly consistent with previous research. In older patients with an urgency component, coping strategies tracked symptom severity rather than age [15]. Another study found that age was associated with lower self-perceived UI severity, and with the use of fewer self-management strategies overall, although associations between age and specific behavioural modifications, such as beverage intake, were less evident. Also, women who attributed their incontinence to ageing were less likely to restrict fluid intake [16].

It is worth mentioning the hormonal reasons for changes in fluid regulation, as older patients excrete water more slowly and are at higher risk of hyponatremia, as sex steroid changes across the menopause regulate cardiovascular function, arterial tension and sodium load [17]. That our older participants reported less restriction suggests that it might be driven more by social exposure to work or the physiological vulnerability to consequences of underdrinking.

The dietary results require more caution than the primary finding. Fruits are frequently cited as bladder irritants associated with a higher frequency of urgency episodes, especially citrus and other acidic fruits. The fruit-SUI association was weak and did not reach the conventional threshold for statistical significance in either the univariable or multivariable analysis. The estimates were similar in direction before and after adjustment, but their imprecision and exploratory nature preclude interpretation as evidence of a directional dietary effect. Even though association did not reach the threshold, the direction seems to be concordant with the recent data. A meta-analysis found healthy dietary patterns associated with fewer incontinence symptoms, with the healthy, plant-rich dietary patterns being effective against lower urinary tract symptoms (LUTS) and urinary incontinence [18]. A Finnish cross-sectional study showed that daily fruit consumption was associated with 24% lower odds of stress-incontinence symptoms, with a higher overall-diet quality revealing the same pattern, whereas frequent consumption of ready-made and fast foods was associated with higher odds [19]. Therefore, we make no definite claim that fruit consumption worsens or improves pelvic floor symptoms.

Further observation points the same way. The eight diet-or-fluid x PFDI-20 item correlations are weak and the same behaviour is associated in opposite directions with different symptoms: more frequent sweetening of beverages accompanies more lower-abdominal pressure, but with lower PFDI A3 and A4 scores. Moreover, after Benjamini–Hochberg correction across the complete 100-test item-level diet-PFDI matrix as a single testing family, no association remained statistically significant. We therefore do not interpret any single coefficient as evidence that a particular food or drink is related to a particular symptom.

The findings from quantitative dietary studies in which nutrient intake was quantified directly offer a more detailed picture of the same question, providing the contextual background rather than evidence arising from the present study. Higher protein intake has been linked to urgency incontinence with no apparent effect of other macronutrients [20], whereas other work found no association of UI with carbohydrate, protein or total fat intake, but a positive association with the ratio of polyunsaturated fat, strongly related to body mass and obesity [6,21].

Our data neither support nor contradict these results. We quantified neither macronutrient intake nor energy and used frequency items to describe habits more than exposures. The absence of any diet-PFDI associations reflects the limits of measurements and sample size, not evidence against the quantitative literature. Menopausal status is a common cause of several associations examined, and the time since menopause did differ between incontinence subtypes in our sample (*p* = 0.041). Sex steroid receptors are expressed throughout the lower urinary tract and the anal canal [22,23,24]. Oestrogen depletion during menopause transition is associated with more common and more severe constipation and functional bowel symptoms [25,26,27]. Systemic menopausal hormone therapy (MHT) with accompanying oestrogen local downregulation has been associated with incident urinary and faecal incontinence [22,28,29]. Studies suggest that MHT may increase matrix metalloproteinase-2 activity, resulting in collagen degradation and loss of connective tissue, leading to vaginal laxity [22]. Although systemic MHT may worsen urinary symptoms, local vaginal oestrogen treatment improved symptoms in urge and stress incontinence and lowered the recurrence of urinary tract infection [30].

Nicotine from smoking is a recognised risk factor for overactive bladder and stress urinary incontinence, plausibly through detrusor stimulation and smoking-related chronic cough [31,32]; however, the small number of smokers in our sample (*n* = 5) precludes firm conclusions.

The clinical significance of these findings should not be overstated. The association between an urgency component and self-reported fluid restriction was pre-specified and adjusted. Therefore, it supports asking women with urgency symptoms directly about deliberate fluid restrictions and advising dietary counselling. It is worth highlighting that the restriction is not supported by any dietary recommendation.

This study has several limitations. First, the cross-sectional design precludes causal inference, and many exploratory corrections raise concerns about chance findings despite Benjamini–Hochberg correction. Second, this was a single-centre convenience sample of 87 women, limiting generalisability and statistical precision, particularly for small or moderate associations. Validated Polish-language instruments were available—in particular the Dietary Habits and Nutrition Beliefs Questionnaire (KomPAN), whose reproducibility has been established in Polish adults [33]—using the instrument in place of bespoke form would have provided methodological value to the study.

Third, dietary and fluid habits were assessed with a custom, non-validated frequency questionnaire, not a validated instrument with quantified intake, with no possibility of detailed estimation of energy or macronutrient intake, which might be associated with recall bias. Because the questionnaire was administered only once, test–retest reliability could not be evaluated. As a descriptive assessment of the internal structure of the four food-frequency items, after aligning their coding direction, Cronbach’s α was 0.41 (bootstrap 95% CI, 0.22–0.54; mean inter-item Spearman’s ρ = 0.16). Because these items assess distinct dietary behaviours rather than a single underlying construct, these values were not interpreted as conventional reliability estimates and the items were not combined into a summary score. Thus, conclusions are limited to behavioural habits, not nutritional exposures and no causal or clinical inference might be drawn from the dietary results. Fourth, exposures and outcomes were self-reported by patients, with UI subtyping based on clinical history and urodynamics in uncertain cases.

Strengths of this study include the use of two validated instruments assessing quality of life and pelvic floor symptoms, same-day gynaecological and ultrasound assessments, and a transparent, hierarchical analysis framework that distinguished the primary analysis from secondary exploratory analyses. The relatively homogeneous sample of women from Lower Silesia, Poland, may have reduced heterogeneity but also limits the generalisability of the findings to more diverse populations.

## 5. Conclusions

In this exploratory cross-sectional study of women with urinary incontinence, the presence of an urgency component was associated with greater self-reported fluid restriction, even after adjustment for age, diabetes, BMI and reported fluid intake. Older age was a factor associated with less fluid restriction.

No individual dietary or fluid item was associated with pelvic floor symptom burden once multiplicity across the complete 100-test matrix was taken into account. The nominally significant item-level correlations were weak and inconsistent in direction across items of the same instrument, and they are reported as hypotheses rather than as findings. Also, they do not support dietary advice of any kind. The one dietary estimate that approached the conventional threshold—less frequent fruit consumption with greater self-perceived stress-incontinence severity—is directionally concordant with recent population data and is posed as a specific question for prospective testing, not as a result of this study.

Older age was also independently associated with less self-reported fluid restriction in the adjusted model, although this secondary association should be interpreted cautiously and does not establish its underlying mechanism. The inverse association with age suggests that fluid restriction behaviour may not simply reflect physiological changes associated with ageing and might be driven by circumstance rather than physiology.

The single clinical implication we draw rests on the primary analysis: women presenting with urgency symptoms should be asked directly whether they limit their fluid intake, because the behaviour is common in that group, is not supported by prospective evidence and carries potential harm.

Because dietary behaviour was assessed with a non-validated instrument that quantifies neither portions nor nutrients, and because it was based on a single-centre convenience sample, these associations should be regarded as preliminary. No cross-sectional study design can establish whether dietary or fluid habits modify pelvic floor symptom burden; the question requires a prospective study using a validated quantitative dietary instrument.

## Figures and Tables

**Figure 1:**
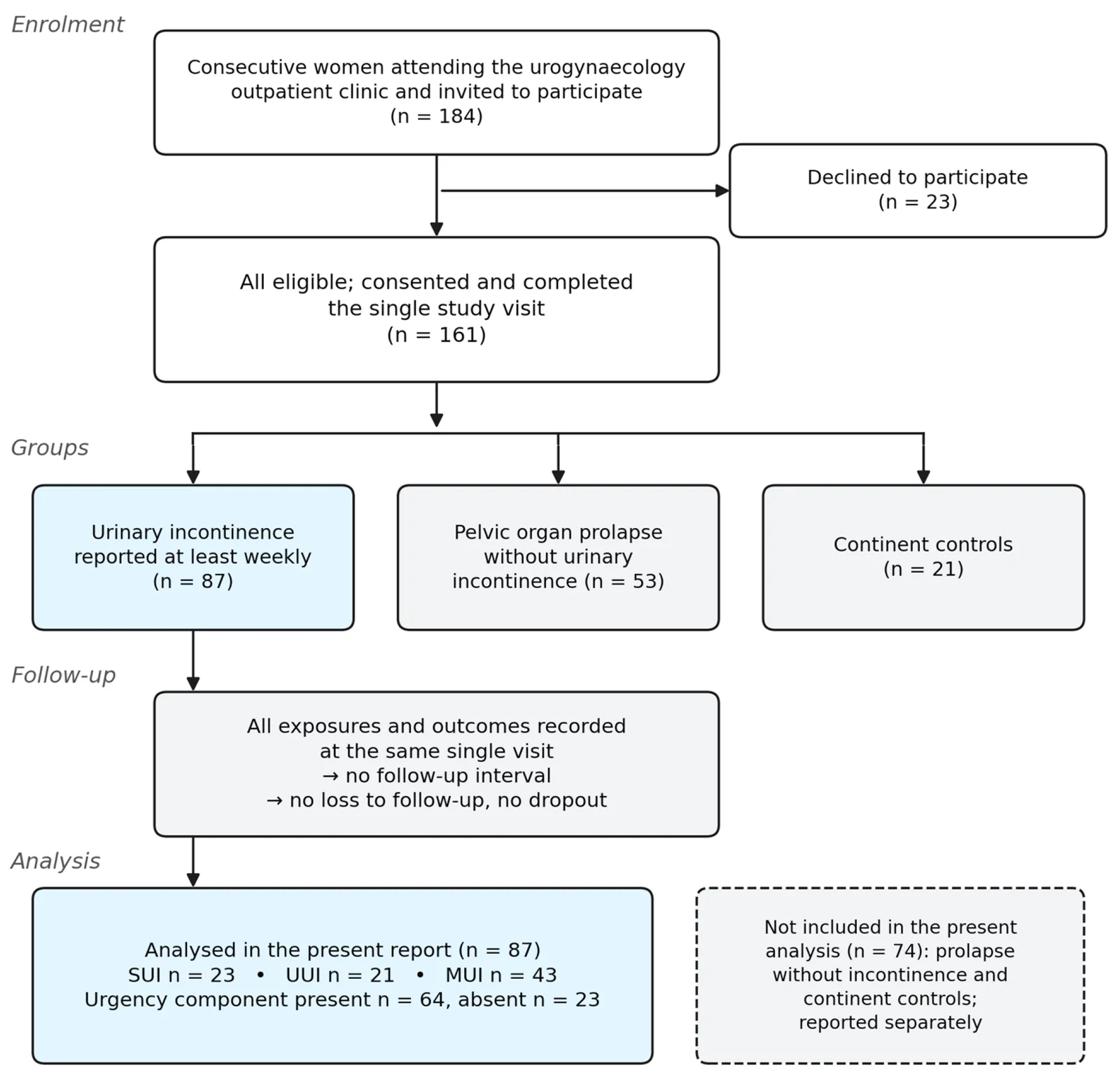
Participant flow diagram. SUI, stress urinary incontinence; UUI, urgency urinary incontinence; MUI, mixed urinary incontinence.

**Figure 2:**
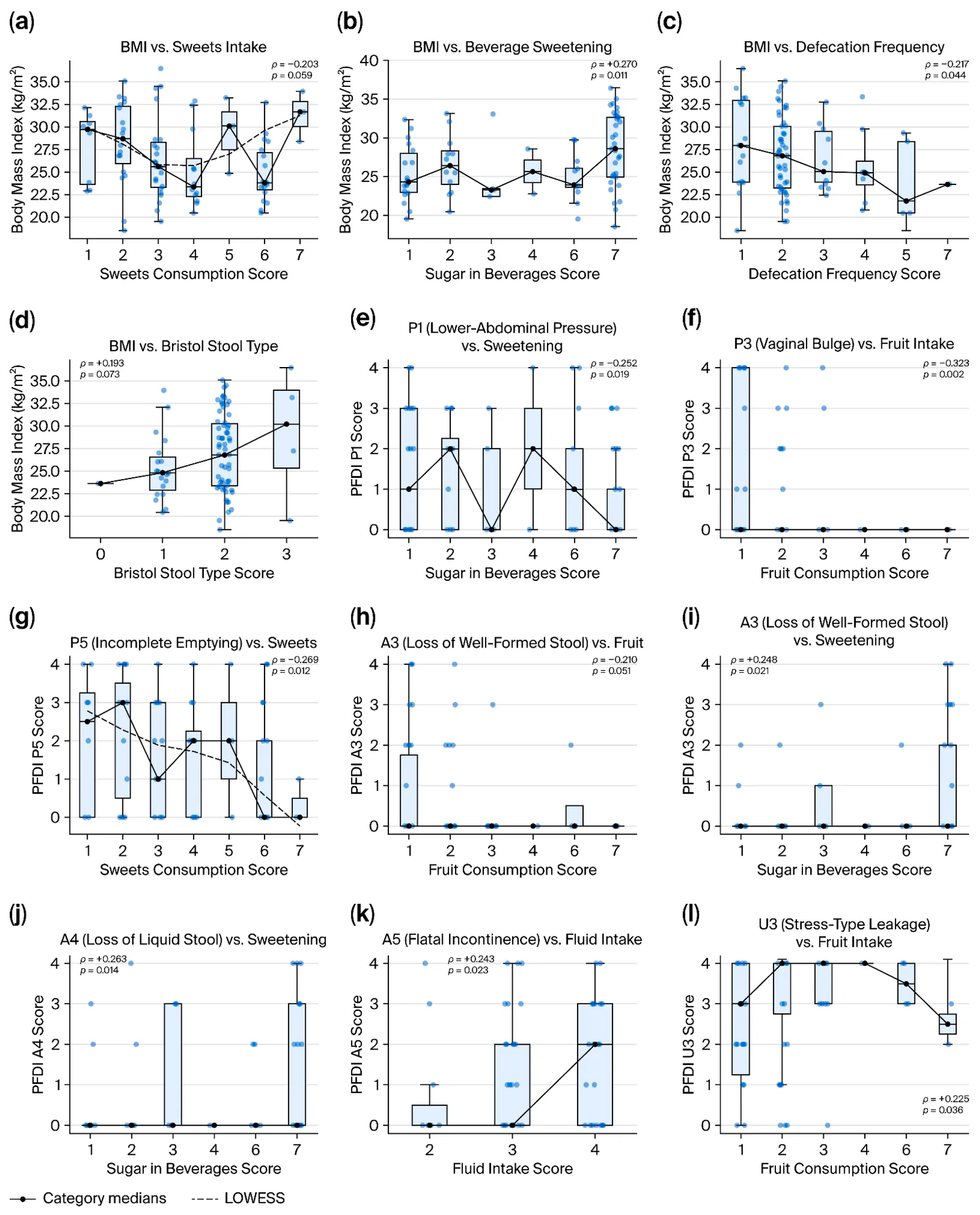
Associations between dietary intake and pelvic floor symptoms or BMI in women with urinary incontinence (*n* = 87): (**a**) BMI versus sweets intake; (**b**) BMI versus beverage sweetening; (**c**) BMI versus defecation frequency; (**d**) BMI versus Bristol stool type; (**e**) PFDI P1 (lower-abdominal pressure) versus beverage sweetening; (**f**) PFDI P3 (vaginal-bulge sensation) versus fruit intake; (**g**) PFDI P5 (incomplete bladder emptying) versus sweets intake; (**h**) PFDI A3 (loss of well-formed stool) versus fruit intake; (**i**) PFDI A3 versus beverage sweetening; (**j**) PFDI A4 (loss of loose stool) versus beverage sweetening; (**k**) PFDI A5 (flatal incontinence) versus fluid intake; and (**l**) PFDI U3 (stress-type leakage) versus fruit intake. Boxes show medians and interquartile ranges, and dots represent individual observations. The solid line connects category medians, and the dashed line is the LOWESS trend. Dietary-frequency scales are shown as collected, with higher scores indicating less frequent intake. ρ denotes Spearman’s rank correlation coefficient; displayed *p*-values are unadjusted (*p* < 0.05). The diet-PFDI associations shown in panels (**e**–**l**) are exploratory; none remained statistically significant after Benjamini–Hochberg correction across the complete 100-test diet/fluid-PFDI matrix.

**Table 1:** Patient characteristics by urinary incontinence subtype.

Characteristic	Total (*n* = 87)	SUI (*n* = 23)	UUI (*n* = 21)	MUI (*n* = 43)	*p*
Age, years—mean ± SD	57.2 ± 13.8	53.0 ± 9.7	60.9 ± 18.5	57.7 ± 12.8	0.069
BMI, kg/m^2^—mean ± SD	26.7 ± 4.3	26.4 ± 4.8	27.9 ± 3.9	26.3 ± 4.2	0.269
Menopausal—*n* (%)	56 (64)	13 (57)	16 (76)	27 (63)	0.378
Years since menopause—median (IQR)	7 (0–17.5)	1 (0–9.5)	17 (1–23)	5 (0–19)	0.041
Parity—median (IQR)	2 (1–2)	2 (2–2)	2 (1–2)	2 (1.5–2)	0.403
Diabetes—*n* (%)	14 (16)	2 (9)	4 (19)	8 (19)	0.530
Hypertension—*n* (%)	25 (29)	2 (9)	11 (52)	12 (28)	0.006
Current smoking—*n* (%)	5 (6)	0 (0)	1 (5)	4 (9)	0.295
Pelvic organ prolapse—*n* (%)	13 (15)	3 (13)	5 (24)	5 (12)	0.420
Prior POP surgery—*n* (%)	13 (15)	3 (13)	4 (19)	6 (14)	0.828
Prior abdominal surgery—*n* (%)	32 (37)	9 (39)	5 (24)	18 (42)	0.359
POPDI-6—mean ± SD	19.9 ± 19.3	11.4 ± 15.3	28.0 ± 23.2	20.5 ± 17.6	0.020
CRADI-8—mean ± SD	19.1 ± 21.3	14.7 ± 14.9	21.1 ± 24.3	20.5 ± 22.8	0.806
UDI-6—mean ± SD	55.2 ± 21.9	43.8 ± 22.0	47.4 ± 20.0	65.1 ± 18.2	<0.001
PFDI-20 total—mean ± SD	94.3 ± 49.3	69.9 ± 43.3	96.5 ± 53.8	106.2 ± 46.3	0.014
I-QOL (impact)—mean ± SD	55.0 ± 23.3	48.3 ± 23.3	55.2 ± 23.0	58.5 ± 23.3	0.273

Data are presented as mean ± SD, median (IQR), or *n* (%). *p*-values were obtained using the Kruskal–Wallis test for continuous variables and the chi-squared or Fisher exact test for categorical variables. Group differences were significant for urinary distress (UDI-6; *p* < 0.001), hypertension (*p* = 0.006), years since menopause (*p* = 0.041), and prolapse distress (POPDI-6; *p* = 0.020) and total pelvic floor distress (PFDI-20 total; *p* = 0.014). The I-QOL value shown is the impact score, for which higher values indicate poorer quality of life. Definitions of diabetes, hypertension, and BMI are provided in Section 2.3.

**Table 2:** Univariable and multivariable proportional-odds regression models for fluid restriction, SUI severity, and grouped OAB severity.

Predictor	Univariable OR (95% CI)	*p*	Multivariable OR (95% CI)	*p*
**A. Fluid restriction (I-QOL item 1.8)**				
Urgency status	2.24 (0.95–5.26)	0.064	2.92 (1.18–7.20)	0.020
Age, per 10 years	0.77 (0.58–1.00)	0.053	0.68 (0.51–0.92)	0.011
Diabetes	1.05 (0.40–2.75)	0.928	1.12 (0.41–3.05)	0.832
Body mass index, per 1 kg/m^2^	0.99 (0.90–1.08)	0.787	1.00 (0.91–1.10)	0.976
Reported fluid-intake score, per 1-point increase	0.70 (0.38–1.30)	0.258	0.59 (0.31–1.13)	0.114
**B. SUI-VAS severity**				
Years since last menstruation, per 10 years	0.69 (0.48–0.98)	0.039	0.62 (0.42–0.91)	0.015
Fruit-frequency score, per 1-point increase	1.31 (1.00–1.73)	0.054	1.35 (1.00–1.82)	0.051
Vegetable-frequency score, per 1-point increase	1.32 (0.91–1.89)	0.139	1.31 (0.90–1.92)	0.159
Sweets-frequency score, per 1-point increase	1.05 (0.84–1.31)	0.670	1.10 (0.86–1.39)	0.450
Beverage-sweetening score, per 1-point increase	0.98 (0.85–1.14)	0.809	1.04 (0.87–1.23)	0.693
Body mass index, per 1 kg/m^2^	0.99 (0.91–1.08)	0.846	1.01 (0.92–1.12)	0.810
Number of deliveries, per 1 delivery	1.11 (0.77–1.58)	0.584	1.02 (0.69–1.50)	0.938
**C. Grouped OAB-VAS severity**				
Vegetable-frequency score, per 1-point increase	1.44 (0.95–2.20)	0.087	1.37 (0.87–2.16)	0.177
Years since last menstruation, per 10 years	1.14 (0.79–1.63)	0.487	1.14 (0.79–1.66)	0.482
Pelvic organ prolapse	0.59 (0.20–1.78)	0.348	0.60 (0.18–1.95)	0.394
Body mass index, per 1 kg/m^2^	1.05 (0.95–1.15)	0.360	1.04 (0.94–1.15)	0.440
Fruit-frequency score, per 1-point increase	1.24 (0.90–1.71)	0.191	1.16 (0.83–1.64)	0.387
Previous abdominal surgery	1.06 (0.46–2.40)	0.897	1.17 (0.49–2.81)	0.723
Number of deliveries, per 1 delivery	1.06 (0.72–1.55)	0.777	1.03 (0.68–1.55)	0.888

ORs are cumulative odds ratios from proportional-odds ordinal logistic regression models. Univariable models include one predictor at a time; multivariable models include all predictors listed within the corresponding panel. All models included *n* = 87 participants. Age and years since last menstruation are expressed per 10-year increase; BMI, dietary-frequency scores, and the reported fluid-intake score are expressed per 1-point increase unless otherwise indicated. Higher dietary-frequency scores indicate less frequent intake. *p*-values are nominal and two-sided.

## Data Availability

The data presented in this study are available from the corresponding author upon reasonable request. The data are not publicly available due to privacy and ethical restrictions.

## Supplementary Materials

The following supporting information can be downloaded at https://www.mdpi.com/article/10.3390/nu18193127/s1, Table S1: STROBE checklist for cross-sectional studies; Table S2: Custom dietary and fluid-intake questionnaire: items, response options and coding. Polish version and the English wording—a forward translation prepared for this report; Figure S1: Correlation heat maps of candidate predictors included in the multivariable models for (a) reported fluid restriction, (b) SUI-VAS severity, and (c) grouped OAB-VAS severity. Cells show pairwise correlation coefficients. Spearman’s rank correlation was used for non-binary pairs and binary-ordinal pairs, point-biserial correlation for binary-continuous pairs, and the phi coefficient for binary-binary pairs. Only the lower triangle of each symmetric correlation matrix is displayed; diagonal self-correlations are omitted.
