# Supplementary material for: Dietary and Fluid-Intake Habits in Patients with Various Types of Urinary Incontinence: A Preliminary Study
Source: Nutrients. 2026 Sep 23;18(19):3127. doi: 10.3390/nu18193127 (PMC13666888; doi:10.3390/nu18193127)
Supplement: Supplementary file 1 [file nutrients-18-03127-s001.zip › nutrients-4481236-supplementary.pdf]

Table S1. STROBE checklist for cross-sectional studies.

No.	Item	Recommendation	Location in manuscript
1a	Title and abstract	Indicate the study design in the title or abstract	Title; Abstract, Methods
1b	Title and abstract	Provide an informative and balanced summary	Abstract
2	Background/rationale	Explain the scientific background and rationale	Introduction, paras 1–5
3	Objectives	State specific objectives, including any prespecified hypotheses	Introduction, final para; Section 2.4
4	Study design	Present key elements of study design early in the paper	Section 2.1
5	Setting	Describe setting, locations, and relevant dates	Section 2.2
6a	Participants	Give eligibility criteria and methods of selection	Section 2.2
7	Variables	Define all outcomes, exposures, predictors, confounders and effect modifiers	Section 2.3
8	Data sources/measurement	Give sources of data and details of methods of assessment	Sections 2.3, 2.4
9	Bias	Describe any efforts to address potential sources of bias	Sections 2.4; Discussion, limitations
10	Study size	Explain how the study size was arrived at	Section 2.4
11	Quantitative variables	Explain how quantitative variables were handled in the analyses	Sections 2.3, 2.4
12a	Statistical methods	Describe all statistical methods, including those used to control for confounding	Section 2.4
12b	Statistical methods	Describe any methods used to examine subgroups and interactions	Sections 2.4, 3.5
12c	Statistical methods	Explain how missing data were addressed	Section 2.4
12d	Statistical methods	Describe analytical methods taking account of sampling strategy	Section 2.4
12e	Statistical methods	Describe any sensitivity analyses	Sections 2.4, 3.5
13a–c	Participants	Report numbers of individuals at each stage; reasons for non-participation; flow diagram	Section 3.1
—	Data completeness	No missing values in any analysed variable (n = 87 throughout)	Section 2.4

14a	Descriptive data	Give characteristics of study participants and information on exposures and confounders	Table 1
14b	Descriptive data	Indicate number of participants with missing data for each variable of interest	Section 2.4; Table S2
15	Outcome data	Report numbers of outcome events or summary measures	Sections 3.1, 3.2
16a	Main results	Give unadjusted and confounder-adjusted estimates and their precision	Table 2
16b	Main results	Report category boundaries when continuous variables were categorised	Sections 2.3, 3.4
16c	Main results	Consider translating estimates of relative risk into absolute risk	Section 3.2
17	Other analyses	Report other analyses done — subgroups, interactions, sensitivity analyses	Sections 3.4, 3.5
18	Key results	Summarise key results with reference to study objectives	Discussion, para 1
19	Limitations	Discuss limitations, including sources of bias or imprecision	Discussion, limitations
20	Interpretation	Give a cautious overall interpretation	Discussion, clinical significance; Conclusions
21	Generalisability	Discuss the generalisability of the study results	Discussion, limitations
22	Funding	Give the source of funding and the role of the funders	Funding statement

Table S2. Custom dietary and fluid-intake questionnaire: items, response options and coding. Polish version and the English wording - a forward translation prepared for this report.

Dietary and fluid items (Part I, section Żywienie)

Item	Polish wording as printed	English translation	Response scale	n per option (codes 1→7)	Frequency direction	Quality direction
1A	Jak często je Pani owoce?	How often do you eat fruit?	Scale A	26 / 36 / 18 / 1 / 0 / 4 / 2	1 = most frequent	1 = most favourable
1B	Jak często je Pani warzywa?	How often do you eat vegetables?	Scale A	35 / 32 / 15 / 2 / 1 / 2 / 0	1 = most frequent	1 = most favourable
1C	Jak często słodzi Pani kawę/herbatę/dodaje cukru do deserów?	How often do you sweeten coffee or tea, or add sugar to desserts?	Scale A (**printed in reverse order**)	18 / 12 / 5 / 2 / 0 / 13 / 37	1 = most frequent	1 = least favourable
1D	Ile płynów wypija Pani dziennie (wszystko — woda, kawa, herbata, soki)?	How much fluid do you drink per day (everything — water, coffee, tea, juice)?	Scale B	0 / 11 / 49 / 27 (codes 1→4)	4 = greatest intake	4 = most favourable
1E.1	Jak często je Pani słodycze?	How often do you eat sweets?	Scale A (**printed in reverse order**)	8 / 19 / 23 / 12 / 3 / 19 / 3	1 = most frequent	1 = least favourable
1E.2	Jakie to słodycze?	Which kinds of sweets?	free text, coded chocolate-based (1); sugar-based (2); other (3, 6); not answered (0)	46 / 30 / 4 / 1 / 6	—	—

Values in the “n per option” column are the numbers of participants selecting each option, from code 1 upwards. No participant selected the lowest fluid-intake category. Item 1C was bimodal: 18 participants reported sweetening drinks several times daily and 37 reported never doing so.

Bowel and symptom items (Part I, section Dolegliwości)

Item	Polish wording as printed	English translation	Response format	Distribution of responses	Variable used in the analyses
2A	Nietrzymanie moczu wysiłkowe (np. podczas kaszlu, kichania, śmiania się)	Stress urinary incontinence (e.g. on coughing, sneezing or laughing)	bother, VAS 0–10; frequency, Scale A	VAS median 8 (IQR 5–10), 78 scoring above 0; frequency 51 / 3 / 12 / 2 / 1 / 6 / 11	SUI-VAS
2B	Nietrzymanie moczu związane z parciem na pęcherz (uczucie nagłej, niemożliwej do opanowania potrzeby oddania moczu)	Urgency urinary incontinence (a sudden, uncontrollable need to void)	bother, VAS 0–10; frequency, Scale A	VAS median 7 (IQR 3–9), 70 scoring above 0; frequency 52 / 5 / 5 / 1 / 0 / 7 / 17	OAB-VAS

2C	Nietrzymanie stolca	Faecal incontinence	bother, VAS 0–10; frequency, Scale A	VAS median 0 (IQR 0–0), 12 scoring above 0; frequency 1 / 2 / 4 / 2 / 1 / 4 / 71	not used
2D	Nietrzymanie gazów	Flatal incontinence	bother, VAS 0–10; frequency, Scale A	VAS median 2 (IQR 0–4), 49 scoring above 0; frequency 12 / 5 / 14 / 5 / 0 / 18 / 32	not used
2E.1	Jak często oddaje Pani stolec?	How often do you open your bowels?	Scale C	15 / 48 / 10 / 8 / 5 (codes 1→5)	defecation frequency
2E.2	Jaki jest przeważający charakter stolca?	What is the predominant character of your stool?	Scale D	19 / 63 / 4 (codes 1→3)	stool consistency
2E.3	Który obrazek najbardziej oddaje charakter stolca?	Which picture best matches your stool?	illustrated Bristol Stool Form Scale, types 1–7	8 / 20 / 40 / 14 / 3 / 1 / 0 (types 1→7)	stool consistency
2F	Ból podbrzusza	Lower abdominal pain	bother, VAS 0–10; frequency, Scale A	VAS median 1 (IQR 0–4), 47 scoring above 0; frequency not entered into the dataset	not used
2G	Wrażenie niepełnego oddania moczu	Sensation of incomplete voiding	bother, VAS 0–10; frequency, Scale A	VAS median 2 (IQR 0–6), 57 scoring above 0; frequency 31 / 10 / 9 / 2 / 1 / 14 / 17	not used
2H1.	Wstawanie w nocy, żeby oddać mocz; ile razy w nocy występują mikcje	Getting up at night to void; number of nocturnal voids	bother, VAS 0–10; count; frequency, Scale A′	VAS median 5 (IQR 1–8), 73 scoring above 0; voids median 2 (IQR 1–3, range 0–9); frequency 54 / 13 / 3 / 1 / 0 / 8 / 8	nocturia VAS; nocturnal void count
3A	Czy występuje problem z rozpoczęciem mikcji?	Do you have difficulty initiating micturition?	yes / no	not analysed	not used
3B	Czy występuje problem z oddaniem moczu do końca?	Do you have difficulty emptying the bladder completely?	yes / no	not analysed	not used

Items 2A–2H each record a symptom twice: bother, on a visual analogue scale anchored at 0 and 10 and frequency of occurrence. Frequency distributions are given for codes 1 to 7 in order and exclude a small number of unstated responses. The frequency responses to item 2F were not entered into the dataset. The variable described in the manuscript as stool consistency is item 2E.2; the illustrated Bristol Stool Form Scale (2E.3) was recorded separately and was analysed separately in the BMI-Bristol association shown in Figure 2d. Items 2C, 2D, 2F, 2G, 2E.3, 3A and 3B were collected as part of routine clinical assessment and are listed here for completeness.

Response scales

Scale	Polish wording as printed on the form	Response options and analysis codes (English)

A	kilka razy dziennie; raz dziennie; kilka razy w tygodniu; raz na tydzień lub częściej niż raz na miesiąc; raz na miesiąc; sporadycznie; nigdy	several times a day (1); once a day (2); several times a week (3); once a week, or more often than once a month (4); once a month (5); occasionally (6); never (7)
A′	as Scale A, except: kilka razy w nocy; raz w nocy	as Scale A, except that the two most frequent options are: several times a night (1); once a night (2)
B	1 szklankę = ok. 250 ml lub mniej; 2–3 szklanki = ok. 500–750 ml; 4–6 szklanek = 1000–1500 ml; powyżej 6 szklanek	one glass (about 250 mL) or less (1); 2–3 glasses, about 500–750 mL (2); 4–6 glasses, 1000–1500 mL (3); more than 6 glasses (4)
C	kilka razy dziennie; raz dziennie; raz na 2 dni; raz na 3 dni; rzadziej niż raz na 3 dni	several times a day (1); once a day (2); every 2 days (3); every 3 days (4); less often than every 3 days (5)
D	twardy; pośredni; miękki/płynny	hard (1); intermediate (2); soft or liquid (3)

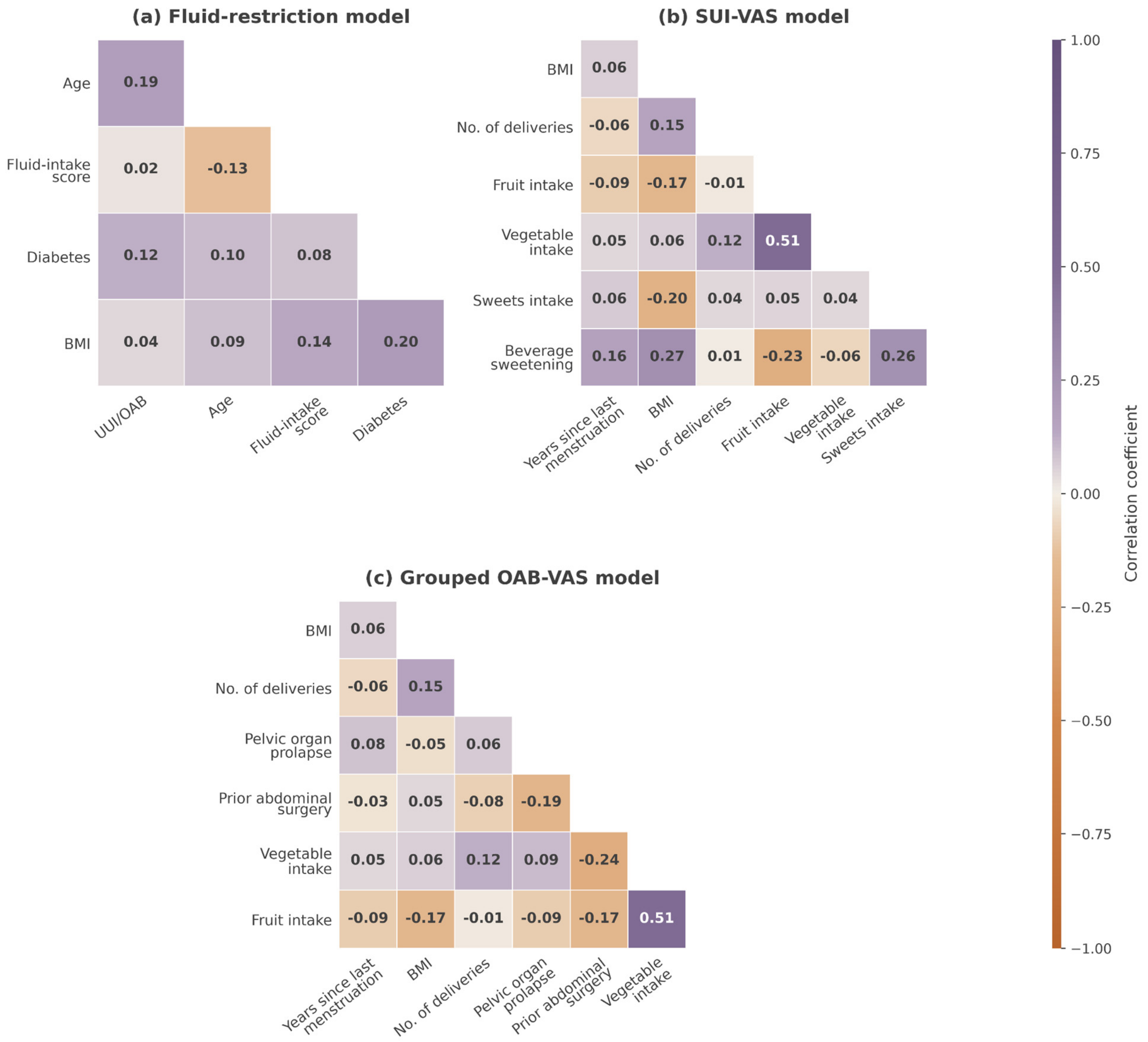

Figure S1. Correlation heat maps of candidate predictors included in the multivariable models for (a) reported fluid restriction, (b) SUI-VAS severity, and (c) grouped OAB-VAS severity. Cells show pairwise correlation coefficients. Spearman's rank correlation was used for non-binary pairs and binary-ordinal pairs, point-biserial correlation for binary-continuous pairs, and the phi coefficient for binary-binary pairs. Only the lower triangle of each symmetric correlation matrix is displayed; diagonal self-correlations are omitted.
